# Retraction Note: Ginkgolide B inhibits lung cancer cells promotion via beclin-1-dependent autophagy

**DOI:** 10.1186/s12906-023-04095-5

**Published:** 2023-07-20

**Authors:** Xuan Wang, Qi-Hui Shao, Hao Zhou, Jun-Lu Wu, Wen-Qiang Quan, Ping Ji, Yi-Wen Yao, Dong Li, Zu-Jun Sun

**Affiliations:** 1 Department of Pharmacy, Putuo People’s Hospital, Shanghai, 200060 China; 2 Department of Traditional Chinese Medicine, Shanghai Tongji Hospital, Tongji University School of Medicine, Shanghai, 200065 China; 3 Department of Clinical Laboratory, Shanghai Tongji Hospital, Tongji University School of Medicine, Shanghai, 200065 China

**Retraction Note: BMC Complementary Medicine and Therapies (2020) 20:194**

10.1186/s12906-020-02980-x

The Editor has retracted this article. After publication, concerns were raised regarding a number of figures in this article. Specifically:

In Fig. 2B, the H1975 and A549 cell images in the 100 mg/l treatment group appear to originate from the same sample with different magnification.

In Fig. 5A, the A549 PCNA western blot image appears highly similar to the NLRP3 blot in Fig. 7C.

In Fig. S2B, the H1975 + Beclin-1-Si-3 DMSO image appears to originate from the same sample as Fig. 2B A549 50 mg/l treatment group with different magnification.

The authors have provided the raw data to address these concerns. However, the raw data files contained further image overlap. The Editor therefore no longer has confidence in the presented data.

All authors agree to this retraction.

